# Rare Case of Enteric *Ancylostoma caninum* Hookworm Infection, South Korea

**DOI:** 10.3201/eid2601.191335

**Published:** 2020-01

**Authors:** Bong-Kwang Jung, Jung-Yeop Lee, Taehee Chang, Hyemi Song, Jong-Yil Chai

**Affiliations:** Korea Association of Health Promotion, Seoul, South Korea (B.-K. Jung, T. Chang, H. Song, J.-Y. Chai);; Korea Association of Health Promotion, Jeonju, South Korea (J.-Y. Lee); Seoul National University College of Medicine, Seoul (J.-Y. Chai)

**Keywords:** *Ancylostoma caninum*, dog hookworm, colonoscopy, South Korea, enteric infections, parasites

## Abstract

A 60-year-old man from South Korea underwent a colonoscopy. A juvenile female worm showing 3 pairs of teeth in the buccal cavity was recovered from the descending colon. Partial sequencing of the internal transcribed spacer region showed 100% identity with *Ancylostoma caninum*, the dog hookworm.

*Ancylostoma duodenale* and *Necator americanus* are the 2 major hookworm species causing human enteric infections around the world. However, *Ancylostoma ceylanicum*, which infects mainly dogs and cats, has become an emerging hookworm species causing human enteric infections in Southeast Asia and the Pacific Islands (*1*,*2*). Another hookworm species, *Ancylostoma caninum*, which infects dogs and rarely cats, can also infect humans, possibly as cutaneous larva migrans (*3*) or enteric infections in association with eosinophilic enteritis (*4*–*6*). Human enteric *A. caninum* infection has been known mainly in Australia (*4*,*5*). However, 2 sporadic human cases (serologically positive but not based on recovery of worms) were reported in the United States (*6*), and samples from 3 schoolchildren in South Africa were detected molecularly to be egg positive (*7*).

We recently encountered a case of *A. caninum* infection in a 60-year-old man who visited a health checkup center and demonstrated a moderate degree of eosinophilia. During colonoscopy, a nematode parasite (female) was found and removed with forceps. The worm was then morphologically and molecularly confirmed to be a dog hookworm, *A. caninum*.

The patient had no subjective symptoms such as abdominal pain, diarrhea, dark feces, or weight loss. However, laboratory examinations showed some abnormalities in liver functions and blood values: total bilirubin 2.11 mg/dL (reference range 0.2–1.57 mg/dL), gamma-glutamyltransferase 238 IU/L (reference range 0–56 IU/L), total serum IgE 1,265 IU/mL (reference range 0–87 IU/mL), and peripheral blood eosinophilia 7.5% (reference range 0%–0.45%). Other blood results were within normal limits: hemoglobin 14.6 g/dL, lymphocytes 26.6%, monocytes 8.0%, basophils 0.9%, and neutrophils 57.0%. The patient owned a dog for 5 years and had regular contact with the dog but had never traveled abroad.

Colonoscopy showed a single threadlike moving worm at the descending colon (Figure, panels A, B). Under magnifying endoscopy, the worm looked like a nematode hooking its head into the colonic mucosa. We removed the worm and sent it to the laboratory. The worm was cleared in lactophenol before microscopic examinations and was found to be a juvenile female hookworm, 12 mm in length and 0.36 mm in width, morphologically identified as *A. caninum* based on the presence of its characteristic 3 pairs of teeth in the buccal cavity (Figure, panel C). We treated the patient with a 3-day course of albendazole.

**Figure:**
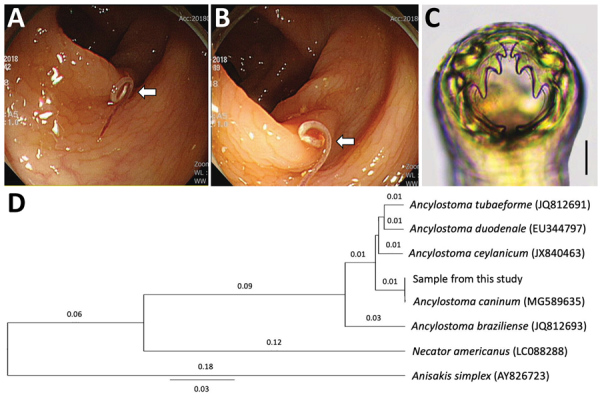
Analysis of a worm from a 60-year-old man in South Korea that was identified as *Ancylostoma caninum*, the dog hookworm. A, B) Colonoscopy images showing a moving threadlike nematode in the mucosa of the descending colon. This nematode (arrows) is seen hooking its head into the colonic mucosa. C) The head part of the worm, showing its characteristic morphology of 3 pairs of teeth in the buccal cavity, by which it could be morphologically identified as *A. caninum*. Scale bar indicates 0.1 mm. D) Phylogenetic tree based on nucleotide sequences of the internal transcribed spacer 1 region and 5.8S rDNA of the worm from this study in comparison with various hookworm species deposited in GenBank (accession numbers indicated in parentheses), inferred by using the UPGMA method. MEGA-X (https://www.megasoftware.net) was used as the software. Scale bar indicates evolutionary distance.

After morphologic examinations, we cut the middle part of the nematode and preserved it in 70% ethanol for molecular studies. We isolated genomic DNA from the worm segment using the DNeasy Blood and Tissue Kit (QIAGEN, https://www.qiagen.com), according to the manufacturer’s instructions. We partially amplified the internal transcribed spacer 1 and 5.8S rDNA regions (312 bp) using the standard PCR protocol with 2x MasterMix (MGmed, https://www.mgmed.com) and 10 pmol of forward and reverse primers to detect *Ancylostoma* species (*8*). The PCR product obtained was directly sequenced at Macrogen Inc. (Seoul, South Korea). The results revealed 100% homology with the sequences of *A. caninum* deposited in GenBank (accession no. MG589635) (Figure, panel D). Our sample also revealed 98.1% homology with *A. duodenale*, 97.1% with *A. ceylanicum*, and 92.4% with *Ancylostoma braziliense*.

Human enteric *A. caninum* infection is extremely rare except in northeastern semitropical regions of Queensland, Australia, where several hundred cases in the form of eosinophilic enteritis have been detected (*5*). Most of these human cases came from a typical suburban environment; in Townsville district, Queensland, 50% of the population have a dog at home, and 69% of adults regularly engage in high-risk behavior, such as walking barefoot on damp grass frequented by dogs (*5*). Human infections with infective-stage larvae appear to occur through a cutaneous route (*5*) or ingestion of infective larvae (*9*).

Signs of human infections include ambiguous abdominal pain that may or may not be associated with eosinophilia (*5*). This patient had no remarkable subjective symptoms except for circulating eosinophilia and markedly elevated total serum IgE. Some abnormalities were noted in liver functions, including slightly elevated total bilirubin and markedly elevated gamma-glutamyltransferase levels. However, these findings seem to have little meaning in relation to *A. caninum* infection.

The worm from this case was a juvenile female that did not contain eggs in its uterus. Thus, our result agrees with previous reports from Australia (*4*,*5*) that these worms do not mature sufficiently to cause infection; they survive probably for only a few weeks. However, recent studies in South Africa (*7*) and India (*10*) detected hookworm eggs in human feces; these were molecularly confirmed to be *A. caninum*. This finding apparently showed that *A. caninum* hookworms can fully develop into egg-producing adults within the human intestine, although occurrence may be rare.

Our study is epidemiologically noteworthy because human enteric *A. caninum* infection may also occur in countries in Asia where this hookworm species is common among dogs, as well as in Australia, the United States, India, and South Africa. Even symptomatic infections with *A. caninum* hookworms may often be missed or misdiagnosed as other conditions or diseases.
